# Computed tomography myocardial perfusion vs ^15^O-water positron emission tomography and fractional flow reserve

**DOI:** 10.1007/s00330-016-4404-5

**Published:** 2016-06-22

**Authors:** Michelle C. Williams, Saeed Mirsadraee, Marc R. Dweck, Nicholas W. Weir, Alison Fletcher, Christophe Lucatelli, Tom MacGillivray, Saroj K. Golay, Nicholas L. Cruden, Peter A. Henriksen, Neal Uren, Graham McKillop, João A. C. Lima, John H. Reid, Edwin J. R. van Beek, Dilip Patel, David E. Newby

**Affiliations:** 1 University of Edinburgh/British Heart Foundation Centre for Cardiovascular Science, Chancellor’s Building, 49 Little France Crescent, Edinburgh, UK EH16 4SB; 2 Clinical Research Imaging Centre, University of Edinburgh, Edinburgh, UK; 3 Edinburgh Heart Centre, Royal Infirmary of Edinburgh, Edinburgh, UK; 4 Department of Radiology, Royal Infirmary of Edinburgh, Edinburgh, UK; 5 Departments of Medicine and Radiology, Johns Hopkins Hospital, Baltimore, MD USA

**Keywords:** Imaging, Perfusion, Ischaemia, Angiography, Angina

## Abstract

**Objectives:**

Computed tomography (CT) can perform comprehensive cardiac imaging. We compared CT coronary angiography (CTCA) and CT myocardial perfusion (CTP) with ^15^O-water positron emission tomography (PET) and invasive coronary angiography (ICA) with fractional flow reserve (FFR).

**Methods:**

51 patients (63 (61–65) years, 80 % male) with known/suspected coronary artery disease (CAD) underwent 320-multidetector CTCA followed by “snapshot” adenosine stress CTP. Of these 22 underwent PET and 47 ICA/FFR. Obstructive CAD was defined as CTCA stenosis >50 % and CTP hypoperfusion, ICA stenosis >70 % or FFR <0.80.

**Results:**

PET hyperaemic myocardial blood flow (MBF) was lower in obstructive than non-obstructive territories defined by ICA/FFR (1.76 (1.32–2.20) vs 3.11 (2.44–3.79) mL/(g/min), *P* < 0.001) and CTCA/CTP (1.76 (1.32–2.20) vs 3.12 (2.44–3.79) mL/(g/min), *P* < 0.001). Baseline and hyperaemic CT attenuation density was lower in obstructive than non-obstructive territories (73 (71–76) vs 86 (84–88) HU, *P* < 0.001 and 101 (96–106) vs 111 (107–114) HU, *P* 0.001). PET hyperaemic MBF corrected for rate pressure product correlated with CT attenuation density (*r* = 0.579, *P* < 0.001). There was excellent per-patient sensitivity (96 %), specificity (85 %), negative predictive value (90 %) and positive predictive value (94 %) for CTCA/CTP vs ICA/FFR.

**Conclusion:**

CT myocardial attenuation density correlates with ^15^O-water PET MBF. CTCA and CTP can accurately identify obstructive CAD.

***Key Points*:**

•*CT myocardial perfusion can aid the assessment of suspected coronary artery disease*.

• *CT attenuation density from “snapshot” imaging is a marker of myocardial perfusion*.

• *CT myocardial attenuation density correlates with* ^*15*^ *O-water PET myocardial blood flow*.

• *CT attenuation density is lower in obstructive territories defined by invasive angiography*.

• *Diagnostic accuracy of CTCA+CTP is comparable to invasive angiography + fractional flow reserve*.

**Electronic supplementary material:**

The online version of this article (doi:10.1007/s00330-016-4404-5) contains supplementary material, which is available to authorized users.

## Introduction

Computed tomography coronary angiography (CTCA) can be performed at low radiation dose and has an excellent negative predictive value in the assessment of coronary artery disease (CAD) [1, 2]. However, areas of high density, such as calcification or stents, can reduce its accuracy. The concomitant assessment of myocardial perfusion could address this issue by providing additive functional information. Computed tomography myocardial perfusion (CTP) has the potential to improve the diagnostic accuracy of CTCA and can be performed during the same examination [3].

The selection of patients for coronary revascularization is based on the functional significance of stenoses. This is best characterised by fractional flow reserve (FFR) which is the current gold standard method of defining obstructive CAD and can lead to improved clinical outcomes when used to guide therapy [4]. However, FFR assessment requires invasive coronary angiography (ICA) and is a derived ratio of pressure rather than a direct measurement of blood flow. Other methods, such as magnetic resonance imaging (MRI) or single photon emission computed tomography (SPECT) can non-invasively assess myocardial perfusion. However, all investigations may be limited by spatial resolution, imaging artefacts, long imaging times and false negatives due to balanced ischaemia in three-vessel disease [5, 6].

Positron emission tomography (PET) using oxygen-15-labeled water (^15^O-water) is the gold standard for the assessment of myocardial perfusion as it is a freely diffusible metabolically inert tracer, with a high extraction fraction that is independent of flow rate [7]. This tracer enables quantitative assessment of absolute myocardial blood flow, rather than relative myocardial blood flow identified by visual assessment in other techniques. ^15^O-Water PET myocardial blood flow measurements have been validated with radiolabelled microspheres in animal models [8, 9] and compared with ICA, ICA/FFR, SPECT, MRI and CTCA in human studies [10–14]. It has good intraobserver and interobserver variability as well as a low inter-scan variability for absolute myocardial blood flow [15, 16].

The diagnostic accuracy of CT myocardial perfusion imaging has been established in multi-centre studies as compared to SPECT [17] and SPECT+ICA [3]. In addition, there have been a number of single-centre studies comparing “snapshot” or “dynamic” CT myocardial perfusion imaging to a variety of gold standard assessments, including FFR [18, 19]. The acceptability of this non-invasive test to patients is also good [20]. However, snapshot CT myocardial perfusion imaging has not previously been assessed as compared to the physiological gold standard of ^15^O-water PET myocardial blood flow. Furthermore, semi-quantitative assessment of CT myocardial perfusion imaging with measurements of attenuation density has not been assessed as compared to either clinical (FFR) or physiological (^15^O-water PET myocardial blood flow) gold standards.

This study assessed snapshot CT myocardial perfusion imaging (also known as single-shot, static and single-phase myocardial perfusion imaging), which obtains a small number of images at maximal contrast enhancement. In order to thoroughly assess this technique we compared it to both the physiological gold standard of absolute myocardial blood flow (measured with ^15^O-water PET) and the clinical gold standard measure of coronary stenosis severity (FFR during ICA).

## Methods

The study was conducted in accordance with the Declaration of Helsinki, with local research ethics committee approval and written informed consent of all participants.

### Study population

Participants had known or suspected CAD and were due to undergo ICA for clinical indications. Exclusion criteria were renal failure (serum creatinine greater than 2.26 mg/dL or estimated glomerular filtration rate less than 30 mL/min), allergy to iodinated contrast, pregnancy or contraindication to adenosine. All patients who met the inclusion and exclusion criteria were invited to participate, and of these 19 % agreed. Cardiovascular risk was assessed using the Framingham 10-year cardiovascular risk score.

### Computed tomography

Participants underwent rest and adenosine stress computed tomography (CT) using a 320-multidetector scanner (Aquilion ONE, Toshiba Medical Systems, Japan). They were asked to refrain from caffeine for 12 h. Participants with a heart rate greater than 65/min were administered intravenous metoprolol (maximum 25 mg). Sublingual glyceryl trinitrate (300 μg tablet) was administered prior to, and removed immediately after, rest imaging.

After acquisition of scout images, patients underwent non-contrast wide volume CT using tube voltage 120 kV, tube current based on body mass index (BMI) and scan range from 2 cm below the carina to the base of the heart using volume sizes of 160, 140, 128, 120, 100 or 80 mm. Participants then underwent resting prospective wide volume CTCA. The detector coverage was based on non-contrast imaging and the acquisition window was based on heart rate (70–80 % of the RR interval for heart rates less than 60/min, 30–80 % of the RR interval for heart rates greater than 60/min or irregular heart rates). Tube voltage and current were based on BMI or scout image attenuation. A triphasic injection of iodinated intravenous contrast (iomeprol, 400 mg iodine/mL; Iomeron 400, Bracco, UK) was administered based on BMI (less than 30 kg/m^2^, 50 mL; 30–39 kg/m^2^, 60 mL; at least 40 kg/m^2^, 70 mL). Bolus triggering started the scan when descending aorta enhancement reached 300 Hounsfield units (HU). Stress imaging was performed 10 min after rest imaging and after 4 min of adenosine (140 μg/(kg/min)). The same tube voltage, tube current and contrast were used as for rest imaging. However, scan range was reduced to cover only the left ventricle, and tube current modulation obtained data over a single heartbeat. A further low dose CT (100 kV, targeted 75 % acquisition) was performed at 3 to 4 min to assess myocardial late enhancement.

Calcium scoring images were reconstructed with filtered back projection (Quantum Denoising Software, QDS+; kernel FC02), and CTCA and CTP images were reconstructed with iterative reconstruction (Adaptive Iterative Dose Reduction 3D, Toshiba Medical Systems, Japan; FC05 or FC03 kernels [21] respectively). From the rest CTCA acquisition, the scanner console software identified the “best phase” and reconstructed this phase and those 10 ms either side. Additional reconstructions at any point in the rest acquisition could be produced as required. For the stress CTCA, images were reconstructed every 5 % of the acquisition which covered the entire RR interval of a single heartbeat. Additional reconstructions at any point in the stress acquisition could be produced as required.

### Oxygen-15-labelled water positron emission tomography

Rest and stress ^15^O-water PET-CT was preformed using a hybrid scanner (128-multidetector Biograph mCT, Siemens Medical Systems, Germany). Attenuation correction CT was performed before rest or before rest and stress imaging. An on-site cyclotron (PETtrace 8, GE Healthcare, UK) produced ^15^O, and a radiowater generator (Hydex Oy, Finland) produced the ^15^O-water bolus. Rest imaging was performed using a target of 500 MBq ^15^O-water (15 s bolus, 2-min saline flush). Dynamic acquisition was performed over 5 min (14 × 5, 3 × 10, 3 × 20 and 4 × 30 s). After suitable decay (approximately 10 min), stress imaging was performed with a further 500 MBq ^15^O-water. Dynamic acquisition was performed during adenosine infusion. Dynamic emission images were reconstructed using the UltraHD algorithm (Siemens Medical Systems, Germany) (zoom 2, matrix 128 × 128, voxels 3.18 × 3.18 × 3 mm).

### Invasive coronary angiography and fractional flow reserve

ICA was performed via radial artery as per standard practice. FFR was assessed for major epicardial vessels with stenosis greater than 50 %, where technically possible. FFR is the ratio between distal coronary pressure and aortic pressure at maximal hyperaemia [4]. Pharmacological stress was induced using adenosine (140 μg/(kg/min)). Obstructive CAD was defined as luminal stenosis of at least 70 % on ICA or FFR less than 0.80.

### Image analysis

Assessment of imaging was blinded to results of other modalities. CT was analysed using a post-processing workstation (Vitrea fX, Vital Images, USA). Two trained observers (MCW, DEN) assessed images separately, and differences were agreed by consensus. The single phase with the fewest artefacts was used to assess CT rest and stress imaging. CTP was assessed as per standard guidelines with adjustment of viewing parameters as required [22]. Visual analysis of CTCA was based on the 19-segment model [1] and CTP on the 17-segment model [23]. Segmental data were consolidated into three territories for per vessel perfusion analysis [23]. Obstructive CAD was defined as stenosis greater than 50 % with corresponding hypoperfusion on CTP.

Semi-quantitative assessment of CTP images was performed by assessing CT myocardial attenuation density at rest and during maximal hyperaemia. CT images were assessed in the short axis, and the apex was excluded. Automatically applied myocardial contours were edited manually as required. Attenuation density was measured in each myocardial segment for endocardium, mid-wall, epicardium and full thickness. Transmyocardial perfusion ratio was calculated as the ratio of segmental subendocardial attenuation divided by mean subepicardial attenuation of the short-axis slice [24].

PET was analysed using dedicated software (Carimas 2.4, Finland) using a single tissue compartment model with correction for perfusable tissue fraction and spillover [15]. Images were reoriented into short-axis and myocardial contours defined on digital subtraction images automatically, with manual adjustment. Myocardial blood flow was analysed for the whole left ventricle and segmental data was consolidated into three territories for per-vessel analysis [23]. Coronary vasodilator reserve was defined as the ratio of peak hyperaemic to resting myocardial blood flow [25]. Total coronary resistance was calculated as mean arterial pressure divided by myocardial blood flow at baseline and hyperaemia [25]. Myocardial blood flow and CT attenuation density were corrected for rate pressure product, calculated from heart rate and blood pressure during imaging.

### Statistical analysis

Statistical analysis was performed using SPSS (version 18 for Mac, IBM). Normally distributed quantitative variables are presented with mean and 95 % confidence interval. Non-normally distributed data are presented with median and interquartile range. Statistical significance was assessed using analysis of variance, Student *t* or Mann–Whitney *U* tests as appropriate. Sensitivity, specificity, positive predictive value (PPV), negative predictive value (NPV) and diagnostic accuracy were calculated on a per-vessel and per-patient basis. Receiver operator characteristic curves were constructed to assess diagnostic accuracy. Correlations were assessed using Pearson’s correlations. Inter- and intraobserver variability was assessed using kappa statistics. A statistically significant difference was defined as a two-sided *P* value less than 0.05.

## Results

### Demographics

CT was performed in 51 patients (Table 1). ICA was planned for all patients but only completed in 47 because of either patient or clinician preference. PET was performed in 22 patients who agreed to additional imaging and had not undergone revascularization in the intervening time period. All imaging was therefore performed prior to any revascularization procedure.

**Table 1:** Demographics

*N*	51	
Age (years)	63	(61, 65)
Male/female	41/10	(80/20 %)
Weight (kg)	85.0	(80.0, 90.0)
BMI (kg/m^2^)	28.6	(27.3, 29.9)
Hypertension	36	(71)
Hypercholesterolaemia	48	(94)
Diabetes mellitus	5	(10)
Cerebrovascular disease	4	(8)
Peripheral vascular disease	2	(4)
Current smoker	9	(18)
Ex-smoker (>1 month)	23	(45)
Family history	24	(47)
Previous acute coronary syndrome	13	(25)
Previous revascularisation	13	(25)
Stent	12	(24)
Coronary artery bypass graft	1	(2)
Invasive coronary angiography		
Normal	18	(38)
One-vessel disease	14	(30)
Two-vessel disease	8	(17)
Three-vessel disease	7	(15)

Mean (95 % confidence interval) or number (%)

According to the Framingham 10-year cardiovascular risk score, 23 (45 %) were high risk, 20 (39 %) intermediate risk and 8 (16 %) low risk. Thirteen patients had previously experienced an acute coronary syndrome. Mean coronary artery calcium score was 870 (580, 1161) Agatston units. ICA/FFR identified obstructive CAD in 27/47 (57 %) patients and 51/141 (36 %) of vessels. Participants who underwent PET had similar demographic details and risk factors compared to the other participants (Supplementary Table i).

### Radiation

Mean dose length product (DLP) for CT was 641.44 (558.50–724.24) mGy cm. Using the 0.014 mSv/(mGy cm) conversion factor, we calculated the dose of rest CT as 2.73 mSv, stress CT 3.71 mSv and total CT protocol 8.98 mSv. Mean administered activity of ^15^O-water was 474 (461–488) MBq for rest and 465 (448–482) MBq for stress imaging. Mean DLP of attenuation correction CT was 73 mGy cm. According to the conversion factors of 0.014 mSv/(mGy cm) and 1.1 μSv/MBq [26], this equates to a total effective dose of 2.05 mSv for PET.

### Assessment of myocardial perfusion

^15^O-Water PET demonstrated that the mean myocardial blood flow and myocardial blood flow corrected for rate pressure product for all segments was higher during hyperaemia (2.52 (2.40–2.64) and 2.65 (2.53–2.77) mL/(g/min) respectively) compared to baseline (0.80 (0.76–0.81) and 0.82 (0.79–0.85) mL/(g/min) respectively; *P* < 0.001 for both).

**Fig. 1:**
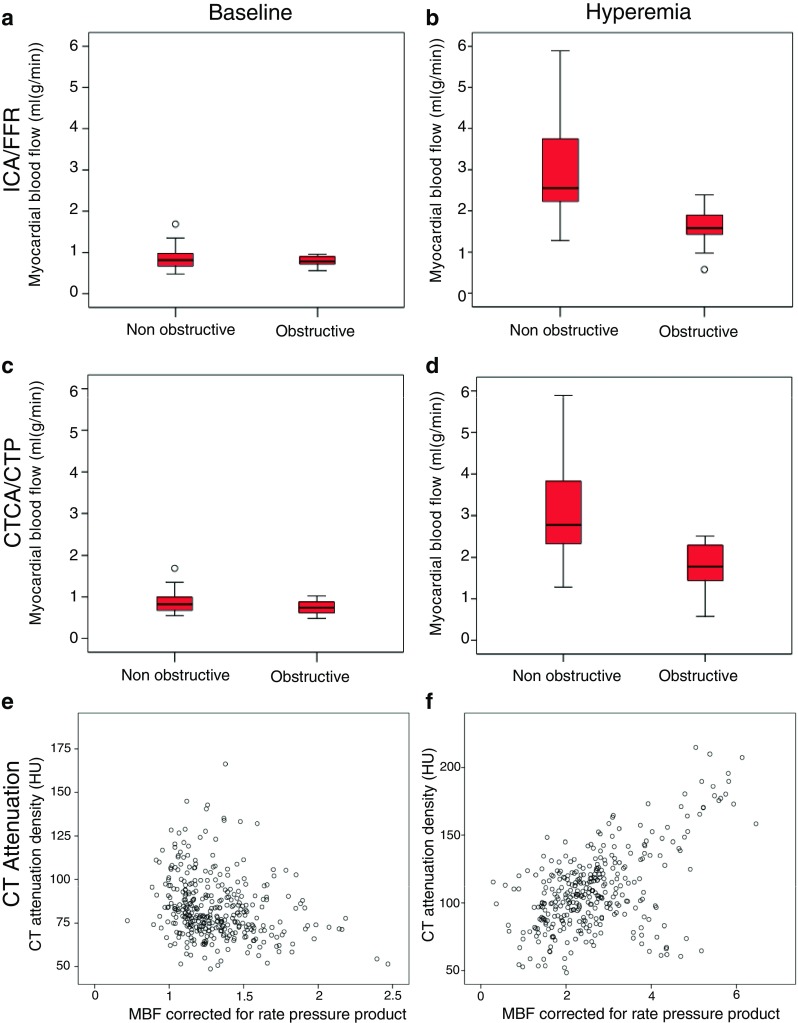
Assessment of myocardial blood flow by ^15^O-water positron emission tomography as compared to computed tomography coronary angiography (CTCA) and myocardial perfusion (CTP) and invasive coronary angiography (ICA) with fractional flow reserve (FFR). Baseline and hyperaemic myocardial blood flow in obstructive and non-obstructive vessels as defined by **a**, **b** ICA/FFR and **c**, **d** CTCA/CTP. **e**, **f** Correlation between myocardial blood flow corrected for rate pressure product and CT contrast enhancement at rest and during hyperaemia respectively

There were no differences in baseline ^15^O-water PET myocardial blood flow between obstructive and non-obstructive lesions as determined by ICA/FFR (*P* = 0.74, Table 2, Fig. 1). However, during hyperaemia, ^15^O-water PET myocardial blood flow was lower for obstructive lesions defined by ICA/FFR on both per-patient and per-vessel assessment (Table 2, Fig. 1).

**Table 2:** ^15^O-Water PET myocardial blood flow (mL/(g/min)) for obstructive and non-obstructive regions as defined by ICA/FFR and CTCA/CTP

		Non-obstructive		Obstructive		*P*
ICA/FFR						
Per patient	Rest	0.88	(0.74, 1.03)	0.63	(0.35, 0.89)	0.074
	Stress	3.11	(2.44, 3.79)	1.76	(1.32, 2.20)	0.001
Per vessel	Rest	0.84	(0.77, 0.91)	0.78	(0.69, 0.88)	0.3
	Stress	2.92	(2.61, 3.23)	1.60	(1.28, 1.91)	<0.001
CTCA/CTP						
Per patient	Rest	0.88	(0.74, 1.03)	0.63	(0.35, 0.90)	0.74
	Stress	3.12	(2.44, 3.79)	1.76	(1.32, 2.20)	0.001
Per vessel	Rest	0.86	(0.79, 0.94)	0.75	(0.67, 0.84)	0.45
	Stress	3.04	(2.70, 3.39)	1.78	(1.51, 2.05)	<0.001
Per segment	Rest	0.79	(0.76, 0.82)	0.65	(0.61, 0.70)	<0.001
	Stress	2.77	(2.63, 2.91)	2.09	(1.93, 2.24)	<0.001

Mean (95 % confidence interval)

**Fig. 2:**
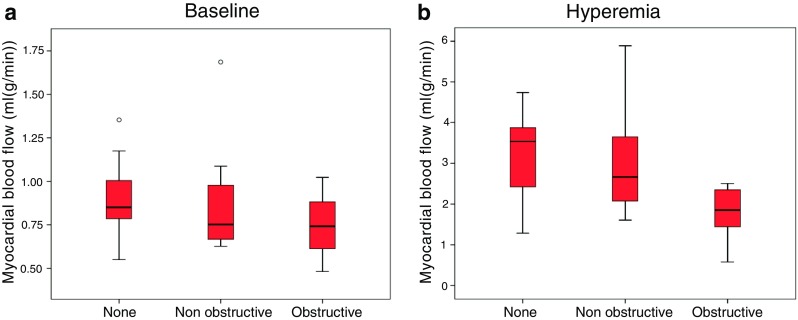
Baseline (**a**) and hyperaemic (**b**) myocardial blood flow assessed by ^15^O-water positron emission tomography in normal, non-obstructive and obstructive vessels as defined by computed tomography coronary angiography and myocardial perfusion

There were no differences in baseline ^15^O-water PET myocardial blood flow in territories supplied by arteries with obstructive or non-obstructive lesions as defined by CTCA/CTP on per-patient or per-vessel analysis (Table 2, Fig. 1). However, on per-segment analysis, baseline ^15^O-water PET myocardial blood flow was lower in territories supplied by coronary arteries with obstructive lesions (0.65 versus 0.79 mL/(g/min), *P* < 0.001). Hyperaemic ^15^O-water PET myocardial blood flow was lower in territories supplied by arteries with obstructive lesions defined by CTCA/CTP on per-patient, per-vessel and per-segment analysis (Table 2, Fig. 1). In addition, vessel territories defined as normal by CTCA/CTP had a higher baseline and hyperaemic ^15^O-water PET myocardial blood flow than those with non-obstructive or obstructive lesions, but this difference was not statistically significant (Fig. 2).

For per-vessel assessment, the optimal cut-off for ^15^O-water PET myocardial blood flow for assessing the presence of obstructive stenosis as defined by either ICA/FFR or CTCA/CTP was 0.52 mL/(g/min) at baseline and 0.78 mL/(g/min) during hyperaemia (Table 3, Supplementary Table ii). The area under the curve was higher for hyperaemic than baseline myocardial blood flow.

**Table 3:** Optimal cut-off value for hyperaemic myocardial blood flow (MBF) on per-vessel assessment to identify obstructive stenosis as defined by ICA/FFR or CTCA/CTP

		Cut-off value (mL/(g/min))	Area under the curve		Sensitivity (%)	Specificity (%)
			(95 % CI)	*P* value		
ICA/FFR	Hyperaemic MBF	0.78	0.897 (0.815, 0.979)	<0.001	100	92
	CFR	1.02	0.880 (0.773, 0.986)	<0.001	100	100
CTCA/CTP	Hyperaemic MBF	0.78	0.856 (0.761, 0.951)	<0.001	100	95
	CFR	1.02	0.880 (0.773, 0.986)	0.02	100	94

Mean CT myocardial attenuation density for all segments was 85 (83–87) HU at baseline and 111 (107–114) HU during hyperaemia (*P* < 0.01). The CT transmyocardial perfusion ratio (TPR) was higher at baseline than hyperaemia (1.68 (1.61–1.76) versus 1.17 (1.13–1.21), *P* < 0.001).

CT myocardial attenuation density was lower in territories supplied by arteries with obstructive as compared to non-obstructive lesions as defined by ICA/FFR (Table 4). ^15^O-Water PET myocardial blood flow correlated with CT myocardial attenuation density (Table 5). The correlation was greater for the endocardium and when corrected for rate pressure product or left ventricular enhancement (Table 5). ^15^O-Water PET myocardial blood flow corrected for rate pressure product correlated weakly with CT myocardial attenuation density at rest (*r* = 0.19, *P* < 0.001) but correlated well during hyperaemia (*r* = 0.579, *P* < 0.001, Fig. 1). CT transmyocardial perfusion ratio correlated only weakly with ^15^O-water PET myocardial blood flow. However, ^15^O-water PET defined coronary vasodilator reserve and total coronary resistance correlated more strongly with CT myocardial attenuation density.

**Table 4:** Myocardial contrast enhancement for obstructive and non-obstructive segments as defined by ICA and FFR

	Non-obstructive		Obstructive		*P*
Myocardial contrast enhancement (HU)					
Rest	86	(84, 88)	73	(71, 76)	<0.001
Stress	111	(107, 114)	101	(96, 106)	0.001
Endocardium contrast enhancement (HU)					
Rest	95	(93, 97)	84	(80, 87)	<0.001
Stress	115	(111, 118)	102	(98, 107)	<0.001
CT contrast corrected for left ventricle enhancement (HU)					
Rest	0.17	(0.17, 0.18)	0.15	(0.14, 0.16)	<0.001
Stress	0.37	(0.36, 0.39)	0.30	(0.28, 0.32)	<0.001
CT corrected for rate pressure product (HU)					
Rest	90	(86, 93)	81	(76, 86)	0.004
Stress	124	(114, 134)	118	(108, 127)	0.330
Transmyocardial perfusion ratio					
Rest	1.62	(1.54, 1.71)	1.91	(1.69, 2.13)	<0.001
Stress	1.23	(1.19, 1.28)	0.98	(0.93, 1.04)	<0.001

Mean (95 % confidence interval)

**Table 5:** Correlation between oxygen-15 PET myocardial blood flow and computed tomography myocardial perfusion imaging at rest and during adenosine stress: for PET parameters as compared to CT myocardial attenuation density; and for CT parameters as compared to oxygen-15 PET myocardial blood flow corrected for rate pressure product

	Baseline	Hyperaemia
PET parameters vs CT myocardial attenuation density		
PET total coronary resistance	0.278, <0.001	0.274, <0.001
PET myocardial blood flow	−0.227, <0.001	0.230, <0.001
Change in PET myocardial blood flow	0.307, <0.001	0.278, <0.001
PET coronary vasodilator reserve	0.411, <0.001	0.460, <0.001
PET myocardial blood flow corrected for rate pressure product	−0.235, <0.001	0.553, <0.001
CT parameter vs oxygen-15 PET myocardial blood flow		
CT attenuation density	−0.235, <0.001	0.553, <0.001
Endocardium CT attenuation density	0.328, <0.001	0.601, <0.001
CT attenuation density corrected for left ventricular enhancement	−0.136, 0.01	0.269, <0.001
CT attenuation density corrected for rate pressure product	0.19, <0.001	0.579, <0.001
Transmyocardial perfusion ratio	0.172, 0.0002	0.214, <0.001

*r* value, *P* value

### Diagnostic accuracy of perfusion imaging

**Fig. 3:**
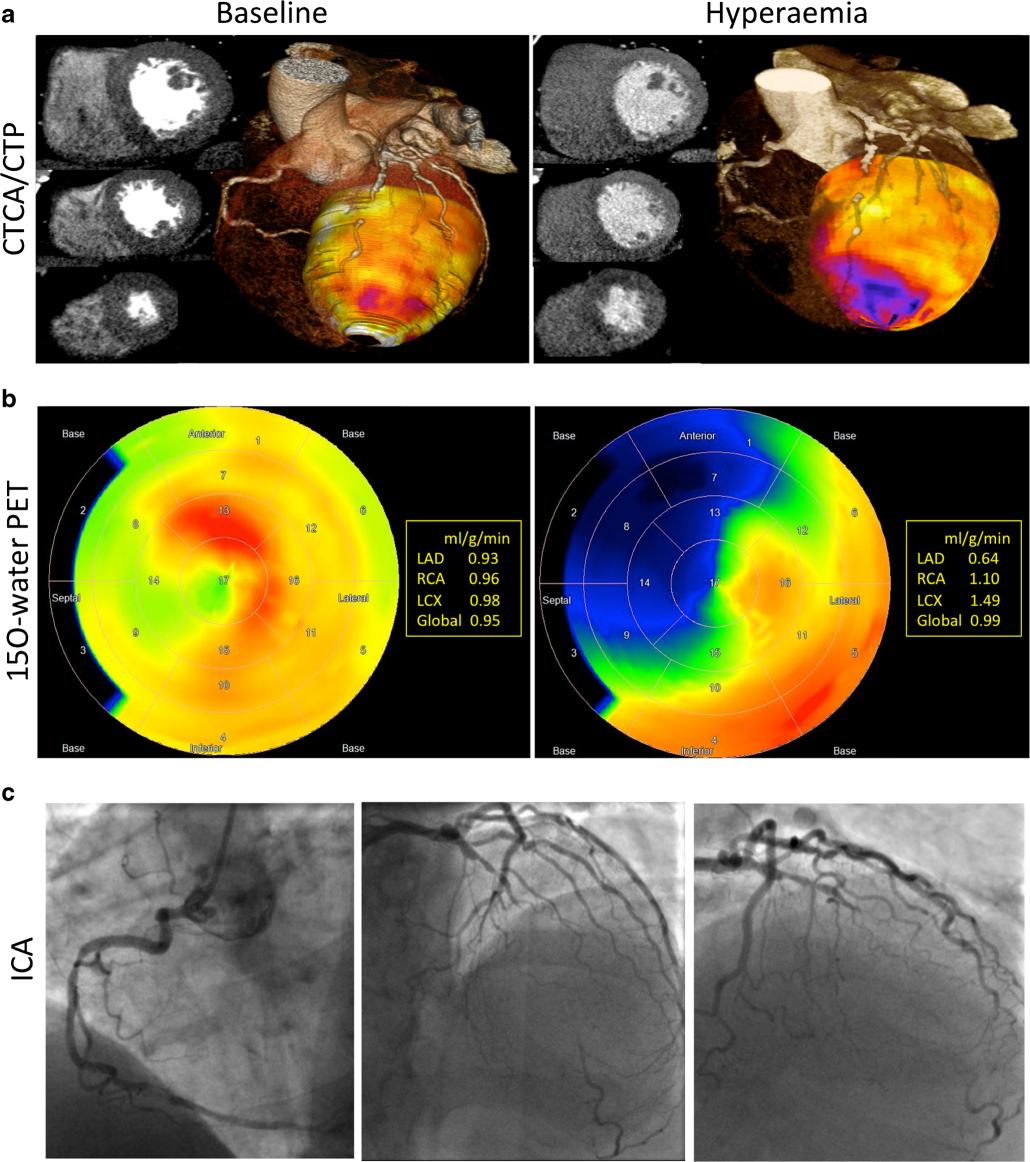
Example images from CT, PET and ICA. This 74-year-old non-smoker with hypertension, hypercholesterolaemia and atypical chest pain underwent multimodality assessment of his cardiac anatomy and physiology. **a** Short-axis basal, mid and apical images of the left ventricle at rest and during hyperaemia along with three-dimensional representations of the transmyocardial perfusion ratio and coronary anatomy. Hypoattenutation is seen in right coronary artery (RCA) and left anterior descending (LAD) artery during hyperaemia. There is also mild hypoattenuation in the RCA territory on rest imaging. **b** Corresponding ^15^O-water PET images which identified the same perfusion abnormalities in terms of the absolute myocardial blood flow (mL/(g/min)). **c** Corresponding images from ICA which identified an occluded LAD and severe stenosis of the RCA with FFR less than 0.80

When compared to ICA/FFR, the sensitivity, specificity, PPV and NPV of CTCA/CTP were 96, 85, 90 and 94 % on a per-patient basis and 88, 83, 75 and 93 % on a per-vessel basis (Supplementary Table iii). Inter- and intraobserver variabilities for CT were good (kappa value 0.772, *P* < 0.001 and 0.721, *P* < 0.001) and for PET were excellent (kappa value 0.817, *P* < 0.001 and 0.942, *P* < 0.001). Figure 3 shows example images. Late enhancement was identified on one CT in a patient with a previous myocardial infarction, and this was confirmed on subsequent MRI. This shows that the majority of perfusion defects identified represent reversible ischaemia rather than infarction.

## Discussion

We have assessed combined CT coronary angiography and snapshot CT myocardial perfusion imaging and have undertaken simultaneous comparisons with the gold standard measure of absolute myocardial blood flow (^15^O-water PET) and the gold standard measure of coronary stenosis severity (FFR during invasive coronary angiography). We have demonstrated for the first time that CT attenuation density correlates with ^15^O-water PET myocardial blood flow. In addition, CT perfusion identified clinically significant CAD as defined by FFR with excellent diagnostic accuracy. Thus we provide physiological and clinical evidence to support the use of CT myocardial perfusion imaging.

CTCA is an established technique for the non-invasive assessment of CAD. However, CTCA alone fails to identify a significant proportion of functionally significant lesions compared with ICA/FFR [27]. This is where perfusion imaging has an important discriminatory role and is additive to CTCA [28]. CORE-320 showed that the CTP and CTCA has a sensitivity, specificity, PPV and NPV for detecting greater than 50 % ICA stenosis with SPECT perfusion defects of 80, 74, 65 and 86 % on a per-patient basis [3]. However, although SPECT is widely used as the first-line perfusion imaging technique by many clinicians, it has limitations [29, 30]. Comparisons with SPECT may under-represent the true value of CTP. We therefore used ICA and FFR as the gold standard clinical reference comparator and confirmed the diagnostic accuracy of CTCA/CTP.

Our study supports the clinical use of CTP in the investigation of patients with suspected CAD. CTP can be used as an optional “add-on” investigation immediately after CTCA when heavily calcified vessels are identified or if the functional severity of stenosis is uncertain. Alternatively, it can be prospectively planned for patients with CAD being considered for coronary revascularisation. In addition to identifying major epicardial CAD, CTP can also identify microvascular dysfunction. In our study, some of the false positives were due to the presence of microvascular dysfunction, highlighted as “slow flow” during ICA with a normal FFR. FFR is primarily focused on identifying a pressure gradient across a functional stenosis but cannot assess microvascular resistance or flow directly [31]. Other reasons for false positive results may include motion artefact or beam-hardening artefact on CT. A high specificity is an important aim for CT myocardial perfusion imaging to enable this to be used to guide referrals for invasive coronary angiography. CT can be used to minimize the number of normal invasive coronary angiographies performed and also provide additional information on anatomy in order to plan revascularization. It is hoped that in the future this could also be used to plan bypass surgery in relevant patients, without the requirement for invasive coronary angiography.

In our study, the optimal cut-off to identify hemodynamically significant stenoses was 0.78 mL/(g/min). Previous studies have reported optimal cut-off values between 1.86 and 2.5 mL/(g/min) [11, 13, 14, 32, 33]. However, our study did not include healthy volunteers and had a high proportion of patients with CAD. Myocardial blood flow is known to be lower in normal regions remote from those with an obstructive stenosis in patients after acute myocardial infarction, as compared to normal controls [25]. Reduced coronary flow velocity reserve has also been identified in women with chest pain without obstructive coronary artery disease on invasive coronary angiography, possibly highlighting microvascular dysfunction [34].

A variety of doses of ^15^O-water have been used in previous studies, including lower doses than used in this study [35, 36]. Although current guidelines recommend a 700–1500 MBq ^15^O-water bolus [37], improved detector sensitivity in the latest generation scanners means that lower doses are appropriate, but may lead to variations in the results. Together, these factors may help explain the lower thresholds identified in our study. The higher ^15^O-water PET myocardial blood flow identified in normal as compared to non-obstructive coronary arteries suggests the potential impact of microvascular disease on myocardial blood flow in patients with non-obstructive coronary artery disease. This difference was not statistically significant in our study but this does warrant further investigation.

This study assessed snapshot CTP where a small number of images are acquired just after the peak of contrast enhancement, rather than dynamic CTP that acquires multiple images during the wash-in and wash-out of contrast. Myocardial blood flow calculated using dynamic CTP correlates with ^15^O-water PET myocardial blood flow [38]. However, dynamic CTP has a higher radiation dose than snapshot CTP. Our study shows that semi-quantitative assessment of snapshot CTP using CT attenuation density also correlates with ^15^O-water PET myocardial blood flow. Previous studies have suggested that rest images alone are sufficient to identify obstructive CAD [39]. However, our data do not support this as rest imaging alone was not sufficient to discriminate between obstructive and non-obstructive lesions.

The use of medications such as glyceryl trinitrate or beta-blockers prior to CTP could alter the presence or severity of perfusion defects [40]. However, previous studies have shown no difference in hyperaemic myocardial blood flow measured with ^13^N-ammonia in patients taking beta-blockers [41]. In addition the diagnostic accuracy of CTCA/CTP identified in our study was similar to previous studies where medications such as beta-blockers and nitrates were withheld. There is also significant heterogeneity in resting myocardial blood flow between individuals receiving these medications [10, 16, 33]. The number of patients included in this study was small and the overall prevalence of CAD was relatively high. Selection bias may also have been introduced as only 19 % consented to participate in this study. This means that assessment of the additive value of CTP over CTCA was difficult to assess. In addition FFR assessment was only performed in major epicardial vessels with stenosis greater than 50 % where technically possible, which may have led to misclassification. Indeed, it is not always possible in a clinical setting to perform FFR because of vessel size or tortuosity. This is an inherent limitation of this “gold standard”, but nevertheless FFR is widely used clinically to guide patient management. Despite having high inter- and intraobserver variability [15] and good agreement between software packages [35], ^15^O-water PET analysis has limitations including potential problems with bolus delivery, PET resolution, patient motion and suboptimal hyperaemia.

In conclusion, this study assessed CTP in comparison to ^15^O-water PET myocardial blood flow and functional severity of coronary stenosis assessed by fractional flow reserve. We have shown that CTCA/CTP provides a robust physiological and clinical assessment of patients with suspected CAD, with excellent correlation with myocardial blood flow and comparable diagnostic accuracy to current invasive gold standard approaches of ICA and FFR. This is the first study to assess the semi-quantitative assessment of CT myocardial perfusion attenuation density as compared to ^15^O-water PET. We believe that CTCA combined with CTP can now be performed in the clinical assessment of patients with suspected CAD.

## Electronic supplementary material

Below is the link to the electronic supplementary material.

Supplementary Table i (DOCX 15 kb)

Supplementary Table ii (DOCX 14 kb)

Supplementary Table iii (DOCX 14 kb)

## Abbreviations

BMI: Body mass index
CT: Computed tomography
CTCA: Computed tomography coronary angiography
CTP: Computed tomography myocardial perfusion imaging
ECG: Electrocardiogram
FFR: Fractional flow reserve
ICA: Invasive coronary angiography
MBF: Myocardial blood flow
MRI: Magnetic resonance imaging
PET: Positron emission tomography
SPECT: Single photon emission computed tomography
