# Supplementary material for: Computed tomography myocardial perfusion vs 15O-water positron emission tomography and fractional flow reserve
Source: Eur Radiol. 2016 Jun 22;27(3):1114–24. doi: 10.1007/s00330-016-4404-5 (PMC5306314; doi:10.1007/s00330-016-4404-5)
Supplement: Supplementary file 1 — (DOCX 15 kb) [file 330_2016_4404_MOESM1_ESM.docx]

*Supplementary Table i: Demographic details for participants who underwent ^15^O-Water PET*

| *Baseline characteristics* |  |  | *P ** |
| --- | --- | --- | --- |
|  |  |  |  |
| N | 22 |  |  |
|  |  |  |  |
| Age (years) | 62 | (58, 65) | 0.121 |
| Male/Female | 41/10 | (80/20%) |  |
| Body mass index (kg/m^2^) | 29.4 | (27.3, 31.6) | 0.291 |
|  |  |  |  |
| Hypertension | 19 | (86) | 0.061 |
| Hypercholesterolemia | 20 | (91) | 0.571 |
| Diabetes mellitus | 3 | (14) | 0.641 |
| Cerebrovascular disease | 2 | (9) | 0.486 |
| Peripheral vascular disease | 0 | (0) | 0.5 |
| Current smoker | 2 | (11) | 0.394 |
| Ex-smoker (>1 month) | 9 | (47) | 0.394 |
| Family history | 10 | (53) | 0.5 |
|  |  |  |  |
| Previous acute coronary syndrome | 5 | (23) | 0.755 |
| Previous revascularisation | 6 | (27) | 1.0 |
| Stent | 6 | (27) | 0.741 |
| Coronary artery bypass graft | 0 | (0) | 1.0 |
|  |  |  |  |
| Framingham 10-year cardiovascular risk score |  |  |  |
| High | 9 | (41) | 0.135 |
| Intermediate | 7 | (32) |  |
| Low | 6 | (27) |  |
|  |  |  |  |

*(Mean (95% confidence interval) or number (%), * compared to the whole population)*
