# Supplementary material for: Computed tomography myocardial perfusion vs 15O-water positron emission tomography and fractional flow reserve
Source: Eur Radiol. 2016 Jun 22;27(3):1114–24. doi: 10.1007/s00330-016-4404-5 (PMC5306314; doi:10.1007/s00330-016-4404-5)
Supplement: Supplementary file 2 — (DOCX 14 kb) [file 330_2016_4404_MOESM2_ESM.docx]

*Supplementary Table ii: Optimal cut-off value for baseline myocardial blood flow (MBF) on per vessel assessment to identify obstructive stenosis as defined by ICA/FFR or CTCA/CTP*

|  |  | Cut-off value  (mL/min/g) | Area under the curve | | Sensitivity (%) | Specificity (%) |
| --- | --- | --- | --- | --- | --- | --- |
|  |  |  | (95% CI) | P value |  |  |
| ICA/FFR | Baseline MBF | 0.52 | 0.545 (0.368, 0.723) | 0.656 | 98 | 100 |
| CTCA/CTP | Baseline MBF | 0.52 | 0.637 (0.476, 0.797) | 0.116 | 100 | 94 |
