# Supplementary material for: Computed tomography myocardial perfusion vs 15O-water positron emission tomography and fractional flow reserve
Source: Eur Radiol. 2016 Jun 22;27(3):1114–24. doi: 10.1007/s00330-016-4404-5 (PMC5306314; doi:10.1007/s00330-016-4404-5)
Supplement: Supplementary file 3 — (DOCX 14 kb) [file 330_2016_4404_MOESM3_ESM.docx]

*Supplementary Table iii: Diagnostic accuracy of computed tomography coronary angiography and myocardial perfusion compared to the gold standard of invasive coronary angiography and fractional flow reserve*

|  | *N* | *TP* | *TN* | *FP* | *FN* | *Sensitivity* | *Specificity* | *PPV* | *NPV* | *Accuracy* |
| --- | --- | --- | --- | --- | --- | --- | --- | --- | --- | --- |
|  |  |  |  |  |  |  |  |  |  |  |
| Per vessel | 141 | 45 | 75 | 15 | 6 | 88 | 83 | 75 | 93 | 85 |
| Per patient | 47 | 26 | 17 | 3 | 1 | 96 | 85 | 90 | 94 | 92 |

*(N number, TP true positive, TN true negative, FP false positive, FN false negative, PPV positive predictive value, NPV negative predictive value)*
